# Smooth tracking of visual targets distinguishes lucid REM sleep dreaming and waking perception from imagination

**DOI:** 10.1038/s41467-018-05547-0

**Published:** 2018-08-17

**Authors:** Stephen LaBerge, Benjamin Baird, Philip G. Zimbardo

**Affiliations:** 1 Department of Psychology, Stanford University, Stanford, CA 94305-2130 USA; 2 Wisconsin Institute for Sleep and Consciousness, Department of Psychiatry, University of Wisconsin–Madison, Madison, WI 53719 USA; 3 Present Address: Lucidity Institute, https://lucidity.com

## Abstract

Humans are typically unable to engage in sustained smooth pursuit for imagined objects. However, it is unknown to what extent smooth tracking occurs for visual imagery during REM sleep dreaming. Here we examine smooth pursuit eye movements during tracking of a slow-moving visual target during lucid dreams in REM sleep. Highly similar smooth pursuit tracking was observed during both waking perception and lucid REM sleep dreaming, in contrast to the characteristically saccadic tracking observed during visuomotor imagination. Our findings suggest that, in this respect, the visual imagery that occurs during REM sleep is more similar to perception than imagination. The data also show that the neural circuitry of smooth pursuit can be driven by a visual percept in the absence of retinal stimulation and that specific voluntary shifts in the direction of experienced gaze within REM sleep dreams are accompanied by corresponding rotations of the physical eyes.

## Abstract (web summary)

When tracking a moving object, our eyes make smooth pursuit movements; however, tracking an imaginary object produces jerky saccadic eye movements. Here, the authors show that during lucid dreams, the eyes smoothly follow dreamed objects. In this respect, dream imagery is more similar to perception than imagination.

## Introduction

Is the visual imagery of dreams more like perception or imagination? This question has been asked at least since Aristotle but until now has lacked empirical data^1^. Here we perform an objective test of this question by determining the extent to which smooth pursuit eye movements (SPEMs) are observed when an individual tracks a slow-moving visual target during lucid rapid eye movement (REM) sleep dreaming. Humans are typically unable to smoothly track slowly an imagined object in a sustained fashion, exhibiting instead saccadic eye movements^2^. Since SPEMs elicited during visual tracking are known to depend on visual motion signals, they may be used as an objective assessment of visual imagery during REM sleep.

Since the discovery of REM sleep in the 1950s, dreams have been believed to be vivid enough to suppose that the REMs that give the state its name are caused by the dreamer scanning the dream scene. Substantial evidence has shown that the strong version of the scanning hypothesis, which suggests that all REMs are due to tracking dream imagery, is untenable. For example, REMs are observed in fetuses, neonates, pontine cats and congenitally blind subjects despite their lack of sight^3^. However, equally substantial evidence indicates that, in other circumstances, there is a close link between the direction of subjective gaze during dreaming and objective eye movements^4,5^. For instance, the pattern of REMs has been found to be related to the visual imagery and reported gaze direction of REM sleep dreams^6^. Furthermore, REMs precisely track saccadic eye movement signals in lucid dreams^7^ and goal-directed actions in dreams of patients with REM sleep behavior disorder^3^. Furthermore, as during waking perception, REMs are associated with transient modulation of spiking activity in the medial temporal lobe associated with visual-mnemonic processes^8^. Together, this evidence suggests that there are multiple sources of eye movements in REM sleep, a subset of which include correspondence between dreamed gaze direction and eye movements.

In order to directly compare visual eye movement tracking in REM sleep dreaming, waking perception and visuomotor imagination, we designed tracking tasks that could be carried out analogously in all three states. Participants were selected for excellent dream recall and high reported frequency of lucid dreams with demonstrated ability to have lucid dreams in the sleep laboratory. Precise psychophysiological correlations were made possible by participants marking the exact moments of initiation of lucidity and the initiation and completion of the tracking tasks with volitional left–right–left–right (LRLR) eye movement signals^7^. Using this eye movement signaling methodology, individuals who are able to reliably attain awareness that they are dreaming while they are dreaming can remember pre-sleep instructions to carry out experiments and mark the exact time of particular dream events with eye movement signals^7,9–11^. As illustrated by LaBerge^12^ these LRLR eye signals are clearly discernable in the electrooculogram (EOG), enabling precise time-stamping of tasks and events during REM sleep (for replications and extensions see, e.g., refs. ^9,13,14^; for recent implementations see, e.g., refs. ^10,15^).

After making the LRLR signal, participants attempted to carry out one of two tracking tasks: (i) circle tracking and (ii) one-dimensional (1D) movement tracking on the horizontal meridian (see Methods). One participant performed a variant of the circle task by tracking a lemniscate (infinity sign). The tracking protocols were carried out in three conditions: (i) lucid REM sleep dreaming (“dreaming”), (ii) awake with eyes open (“perception”) and (iii) tracking the imagined movement while awake with eyes closed (“imagination”). Additionally, one participant completed a fourth condition in which she imagined tracking the movement during a lucid REM sleep dream (“imagination in dreaming”) after completion of the standard tracking task. Immediately upon awakening, participants were interviewed as to whether they performed the task. If participants reported completing the tracking task during a lucid dream, they filled out a dream report form in which they reported their entire dream (see “Dream report methods and analysis“ in “Methods”; see Supplementary Note 1 for examples of participants' reports). Our data provide evidence that intentional slow tracking of visual motion during REM sleep dreams results in SPEMs that are highly similar to waking perception, suggesting that, in this respect, the visual quality of imagery during REM sleep dreaming is more similar to waking perception than imagination.

## Results

### Lucid REM sleep dream tracking and phenomenological reports

Out of the 7 participants, 6 completed at least one tracking task in their lucid REM sleep dreams: 5 participants completed the tracking task a total of 21 times for the circular tracking task (range 1–7) and 3 participants completed the tracking task a total of 5 times for the 1D horizontal meridian tracking task (range 1–3). The total number of trials and results for each condition and participant is provided in Supplementary Table 2 and Supplementary Table 3. Participants reported completing the tracking task as instructed by tracking the thumb of their dominant hand as perceived in the dream in 27 out of the 27 lucid dream tracking trials. Furthermore, 27 out of the 27 lucid dream tracking trials were accompanied by a dream report which described a subjective experience of performing the task by tracking their visual imagery during dreaming, as judged by two independent raters (inter-rater reliability: 100%) (see “Dream report methods and analysis“ in “Methods”). Examples of participants’ dream reports of the tracking task during their lucid REM sleep dreams are provided in Supplementary Note 1.

**Fig. 1:**
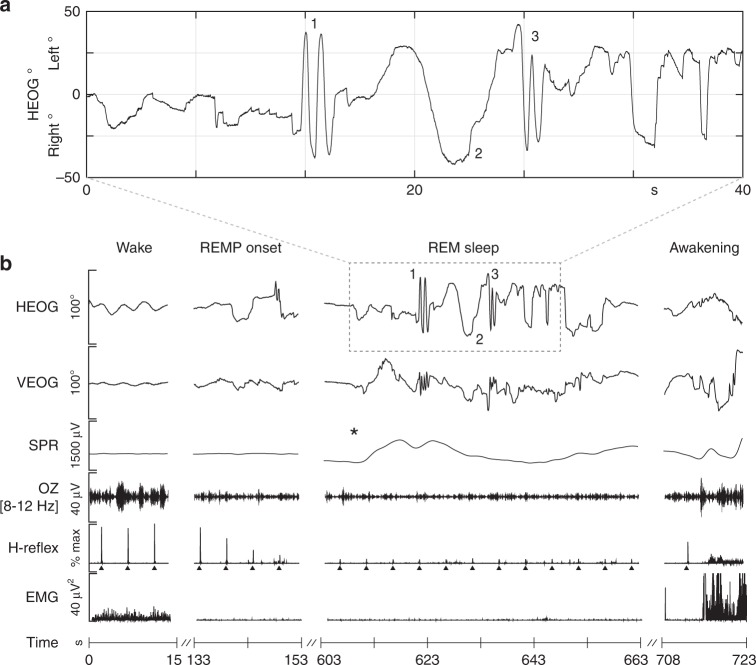
Line tracking during lucid REM sleep dreaming in a single participant. **a** Enlarged section showing LRLR eye movement signals and smooth tracking task as recorded in the horizontal EOG (HEOG). Upon awakening, the subject reported becoming lucid in the dream, making a LRLR signal (1), fully extending his right thumb, and tracking his thumb nail as he slowly swung his arm horizontally from center to approximately 30° left, back through center to 30° right, and finally leftward back to center. While tracking to the right, he noticed moving his head slightly in the direction of tracking both rightward and leftward as he reversed motion back to the center (2). He marked the end of the smooth tracking task (estimated 10 s) with a second LRLR signal (3). Having completed the task, he spent the remainder of his lucid dream exploring the dream environment, waking approximately 60 s later. See Supplementary Note 1 “Report of line tracking task” for the report associated with this figure. **b** Six channels of physiological data (HEOG, vertical EOG (VEOG), skin potential response (SPR)), occipital EEG bandpass filtered for alpha (OZ (8–12 Hz)), H-reflex amplitude (a measure of spinal reflex excitability) (upward black triangles mark H-reflex STIM), and electromyogram (EMG)) are shown during an initial period of wakefulness, REM period onset (REMP onset), transition to lucid REM sleep and awakening. Suppression of EMG and H-reflex amplitude along with reduced alpha in EEG confirm that the participant remained in uninterrupted REM sleep during lucid dreaming, including LRLR signals and during completion of the slow tracking task. Lucid dream onset is localized by the autonomic nervous system surprise response (scalp skin potential response (SPR; black asterisk)) before the LRLR signal

All 27 lucid dream tracking trials occurred during unambiguous REM sleep (see Fig. 1 for a representative example). Suppression of electromyogram (EMG) and H-reflex amplitude along with suppressed alpha in occipital electroencephalography (EEG) confirmed that both LRLR signals and completion of the slow tracking task occurred in uninterrupted REM sleep (Fig. 1b). All 27 signal-verified lucid dreams met the conventional criteria for REM sleep, including activated EEG, episodic REMs and atonic sub-mental EMG. During the experiment, one participant reported a non-lucid dream in which he practiced the circular smooth tracking task (the participant reported dreaming that he was in the laboratory practicing the tracking task but he did not realize he was dreaming until after he woke up). This unique record afforded us an opportunity to compare smooth tracking behavior across lucid and non-lucid REM sleep dreaming (see “Circle tracking”).

### Circle tracking

We observed a main effect of STATE for number of saccades per second (NSPS) (likelihood ratio: 59.03, *P* = 0.00001, two-tailed bootstrap likelihood ratio test). Imagination had a higher saccade rate compared to both perception (*β* = −1.84, *P* *<* 0.00001, 95% confidence interval (CI) (−2.25, −1.48), two-tailed bootstrap test) and dreaming (*β* = −1.36, *P* < 0.00003, 95% CI (−1.81, −0.92), two-tailed bootstrap test) (Supplementary Table 1; Supplementary Figure 1 and 2). No significant difference was observed between perception and dreaming (*β* = 0.48, *P* = 0.14, 95% CI (0.03, 0.94), two-tailed bootstrap test). The smooth pursuit ratio (the amount of time in smooth pursuit divided by the total time spent tracking the movement (SP%)) also revealed a main effect of STATE (likelihood ratio: 66.41, *P* = 0.00001, two-tailed bootstrap likelihood ratio test). Both perception (*β* = 25.26, *P* < 0.00001, 95% CI (20.81, 30.34), two-tailed bootstrap test) and dreaming (*β* = 22.18, *P* < 0.00001, 95% CI (16.28, 27.82), two-tailed bootstrap test) had increased smooth pursuit compared to imagination. No significant difference was observed between perception and dreaming (*β* = −3.10, *P* = 0.50, 95% CI (−9.08, 2.40), two-tailed bootstrap test). SP% was higher for all 5 participants, while NSPS was lower for 4/5 participants during dreaming compared to imagination (Supplementary Table 2).

SP% and NSPS were highly anti-correlated (*r*_s_ = −0.80, *P* < 10^−15^, two-tailed Spearman’s rank-order correlation), indicating that both variables converged in their measurement of pursuit. No significant difference was observed between clockwise and counterclockwise tracking for either NSPS (*β* = −0.11, *P* = 0.68, 95% CI (−0.46, 0.21), two-tailed bootstrap test) or SP% (*β* = 1.15, *P* = 0.75, 95% CI (−3.22, 5.72), two-tailed bootstrap test). As noted above, one participant reported performing one trial of the circle tracking task (LRLR–counterclockwise circle tracking–LRLR) during a non-lucid dream. Both measures of pursuit during this trial (SP%: 90.58; NSPS: 0) were within the 90% prediction interval for the dreaming condition (SP% (68.14, 98.15), NSPS (0, 1.93)), and were outside the 90% prediction interval for the imagination condition (SP% (32.19, 86.51), NSPS (0.30, 4.28)).

### Horizontal vs. vertical eye movements in circle tracking

Both horizontal EOG (HEOG) and vertical EOG (VEOG) distinguished perception and dreaming from imagination for both NSPS and SP% (all *P* < 0.0005, two-tailed bootstrap test). Quantification of the area under the ROC curve (AUC) revealed that HEOG had numerically higher classification accuracy compared to VEOG for both perception (0.93 vs. 0.87 respectively) and dreaming (0.91 vs. 0.83 respectively) contrasted with imagination, though this difference was not statistically significant (all *P* *≥* 0.24, two-tailed bootstrap test, see below). Across all states, HEOG had increased SP% compared to VEOG (all *P* < 0.00001, two-tailed bootstrap test). The fact that HEOG had increased SP% specifically during waking perception suggests that HEOG was more sensitive to SPEMs. This is consistent with other studies that have shown that HEOG is more accurate and less prone to artifact than VEOG^16^. Our second task, 1D horizontal meridian tracking, was therefore designed to isolate the horizontal component in evaluating differences in SPEMs between states.

### 1D horizontal meridian tracking

**Fig. 2:**
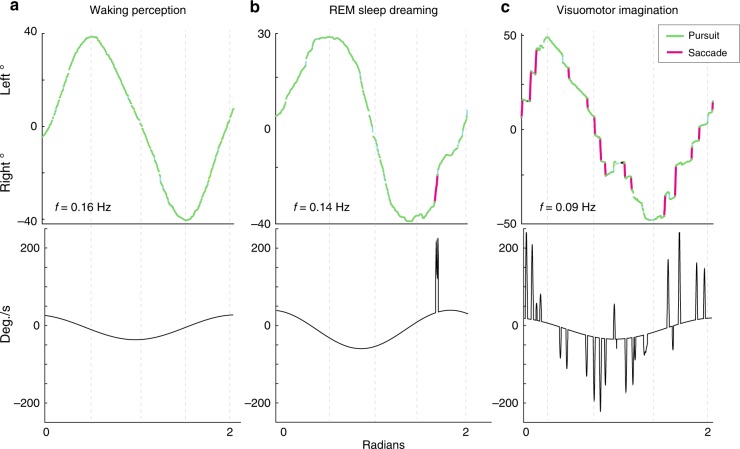
Classification of 1D horizontal meridian tracking for a single participant. Eye-tracking is shown during **a** waking perception, **b** lucid REM sleep dreaming and **c** visuomotor imagination conditions. Slow tracking during imagination showed intrusions of saccadic eye movements during the tracking movement, while waking perception and REM sleep dreaming showed predominately smooth pursuit eye movements. Bottom panels show velocity (degrees/s) as the derivative of the best-fit sine function plus saccades

**Fig. 3:**
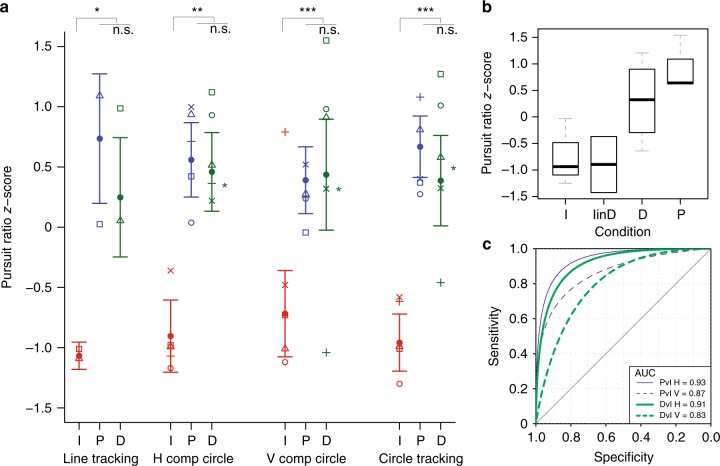
Pursuit ratio scores for perception, dreaming and imagination tracking. **a** Median values of normalized pursuit ratio scores in horizontal line tracking (Line tracking), horizontal (H comp circle), vertical (V Comp Circle) and joint (Circle tracking) components of circle tracking during imagination (I, red line and symbols), perception (P, blue line and symbols) and dreaming (D, green line and symbols). Symbols designate medians for individual subjects. Green stars to the right of D indicate pursuit ratio *z*-score values for the non-lucid REM sleep dream tracking trial. Given that the data were not normally distributed and contained varied numbers of repeated observations within subjects, data were analyzed using a linear mixed model and nonparametric bootstrapping (two-sided, paired test) was used to compare P (circle tracking: *N* = 5, trials = 28; line tracking: *N* = 2, trials = 3), D (circle tracking: *N* = 5, trials = 21; line tracking: *N* = 3, trials = 5) and I (circle tracking: *N* = 5, trials = 28; line tracking: *N* = 2, trials = 3) conditions (replications = 1), *P < 0.001, *P < 0.0001, ***P < 0.00001. Error bars show s.e.m. **b** Boxplot showing pursuit ratio *z*-scores during horizontal line tracking task for imagination (I), imagination in the dream (I in D), perception (P) and dreaming (D) conditions. The bottoms and tops of the boxes show the 25th and 75th percentiles (the lower and upper quartiles), respectively; the inner band shows the median; and the whiskers show the upper and lower quartiles ± 1.5 × the interquartile range (IQR). **c** Area under the ROC curve (AUC) for perception versus imagination (PvI) H (horizontal EOG) (thin blue solid line) and V (vertical EOG) (thin blue dotted line) and dreaming versus imagination (DvI) H (thick green solid line) and V (thick green dotted line) components of eye movements

We observed a main effect of STATE for NSPS (likelihood ratio: 16.85, *P* = 0.017, two-tailed bootstrap likelihood ratio test). Imagination had a higher saccade rate compared to both perception (*β* = −0.93, *P* = 0.0044, 95% CI (−1.26, −0.59), two-tailed bootstrap test) and dreaming (*β* = −0.91, *P* = 0.0029, 95% CI (−1.23, −0.57), two-tailed bootstrap test) (Fig. 2; Supplementary Table 1). No significant difference was observed between perception and dreaming (*β* = 0.01, *P* = 0.868, 95% CI (−0.32, 0.33), two-tailed bootstrap test). SP% also revealed a main effect of STATE (likelihood ratio: 18.32, *P* = 0.01, two-tailed bootstrap likelihood ratio test) (Fig. 3a). Both perception (*β* = 12.63, *P* = 0.003, 95% CI (8.75, 16.57), two-tailed bootstrap test) and dreaming (*β* = 11.35, *P* = 0.0093, 95% CI (7.60, 15.10), two-tailed bootstrap test) had increased smooth pursuit compared to imagination (Fig. 2; Supplementary Table 1). No significant difference was observed between perception and dreaming (*β* = −1.29, *P* = 0.794, 95% CI (−5.11, 2.68), two-tailed bootstrap test).

NSPS was significantly lower for both perception (*β* = −1.27, *P* = 0.006, two-tailed bootstrap test) and dreaming (*β* = −1.25, *P* = 0.009, two-tailed bootstrap test) compared to imagination within the dream, and SP% was significantly higher for both perception (*β* = 31.17, *P* = 0.002, two-tailed bootstrap test) and dreaming (*β* = 28.75, *P* = 0.009, two-tailed bootstrap test) compared to imagination during dreaming (Fig. 3b). For both dreaming and perception, SP% was higher and NSPS was lower for all participants (Supplementary Table 3).

### Bayesian classification of states

SP% as a single indicator yielded 94.7% classification accuracy for perception and 90.0% classification accuracy for dreaming for circle tracking. Bayesian combination of SP% and NSPS increased classification accuracy to 99.6% for perception trials and 98.9% for dream trials (Supplementary Table 4). In the 1D horizontal meridian tracking task, 100% classification accuracy (Bayesian posterior probability (BPP) = 1.00) was achieved for both perception and dreaming (Supplementary Table 5), with an associated sensitivity and specificity of 1.0.

## Discussion

Our data show that intentional slow tracking of visual motion during REM sleep dreams results in SPEMs. Pursuit eye movements in REM sleep did not differ from pursuit during waking perception, and both were characterized by high pursuit ratios and low saccade rates. In contrast, tracking in imagination was characterized by low pursuit with frequent saccadic intrusions. A Bayesian classification model that included pursuit ratio and saccade rate discriminated both REM sleep dreaming and perception from imagination with greater than 98% accuracy. Together, these findings suggest that, at least in this respect, the visual quality of REM sleep dream imagery is more similar to waking perception than imagination.

Evidence suggests that both imagining and dreaming involve activation of the same brain areas as perception in the corresponding sensory modality (see, e.g., refs. ^17–20^). For example, imagining a face or a house activates the same specific brain areas (fusiform face area and parahippocampal place area) involved with perceiving these stimuli^19^. Likewise, dreaming of specific perceptual content, such as faces or speech, recruits overlapping cortical regions as waking perception of these contents^17^. Given these findings, what could explain the reason why perceived and dreamed visual motion are able to drive sustained pursuit while imagined visual motion is not? We hypothesize that it could be attributable to the degree of activation in extrastriate visual areas, particularly the middle temporal visual area (MT) and regions of the superior temporal sulcus that process visual motion. Specifically, evidence suggests that vividness is related to the intensity of neural activation^21,22^ and images, which are typically less vivid, also involve a lesser degree of neural activation than the corresponding perceptions^19^. Furthermore, imagination interferes with perception in the same modality (see, e.g., ref. ^23^), and we may infer the reverse is true as well. In contrast, during REM sleep, sensory input is actively suppressed, preventing competition from externally driven perceptual processes. One interpretation of our results is therefore that under conditions of low levels of competing sensory input and high levels of activation in extrastriate visual cortices (conditions associated with REM sleep), the intensity of neural activation underlying the imagery of visual motion (and therefore its vividness) is able to reach levels typically only associated with waking perception. Sufficient activation of regions such as MT and medial superior temporal areas that are part of the primary pursuit pathway may be needed to drive the pursuit-related motor regions of the cerebellum, such as the flocculus and ventral paraflocculus, which in turn control the output motor nuclei for the eye muscles to engage in sustained pursuit behavior^24^.

Our results help to address a central research question on the topic of smooth pursuit eye movements in humans and non-human primates, which is whether retinal image motion is necessary to drive the neural circuitry of pursuit^2^. While it was initially thought that pursuit required retinal image motion from an external physical motion stimulus, evidence now indicates that smooth pursuit can occur during apparent motion^25^, including motion aftereffects^26^, isoluminant motion^27^ and illusory moving contours^28^, suggesting that SPEMs are linked to the visual percept rather than retinal stimulation^2^. The current results demonstrate that a perceived image with no retinal counterpart (as in the case of the visual imagery during REM sleep) is sufficient to drive sustained smooth pursuit in humans. By demonstrating that smooth pursuit can occur even when there is a complete absence of visual afferent input to the cortex, our findings provide strong evidence that neither a physical motion stimulus nor readout of retinal image motion are necessary for SPEMs.

The current results also provide support for the following form of the scanning hypothesis: intentional shifts in the direction of gaze within a dream are accompanied by corresponding movements of the sleeper’s eyes. Our results show for the first time that tracing circles, lines and infinity signs by tracking visual imagery with one’s eyes in a dream (i.e., the dreamed bodily image of one’s thumb) results in electrooculogram recordings of these shapes. These data provide novel evidence that shifts in the perceived gaze direction in dreams give rise to the appropriate corresponding eye movements. Together, these data are consistent with evidence reviewed above, which suggest that a subset of eye movements during REM sleep are linked to the direction of subjective gaze during dreams.

One limitation of the current study is the small sample size. Only six participants were able to successfully attain lucidity during REM sleep and perform the eye movement tracking task at least once during the study. The limitation in sample size is due to the fact that we worked with an exceptional sample of frequent lucid dream recallers who would be able to reliably attain lucidity and carry out a high-level experimental task in their REM sleep dreams in a sleep laboratory setting. However, despite the relatively small sample, we were able to obtain a substantial number of tracking trials (27) during REM sleep. Furthermore, the fact that our analyses were conducted using mixed models allowed us to analyze data from trials rather than single-subject averages, which allowed us to obtain estimates of effects based on a large number of data points. Nevertheless, studies using larger samples examining these questions would be a desirable goal for future research.

The fact that our study sample was comprised of a highly selected group of frequent lucid dreamers may, on the one hand, be seen as a methodological strength, in line with a long tradition in neuropsychology of gleaning insights about the brain and behavior from the study of special populations. Indeed, without studying this specialized group it is difficult to imagine how this experiment could have been conducted. On the other hand, it might be asked whether, or to what extent, the current results extend generally to (non-lucid) REM sleep dreaming. In the course of data collection, we fortuitously obtained data that directly address this issue. Specifically, during the experiment one participant reported a non-lucid dream in which he practiced the circular smooth tracking task. That is, the participant had a dream in which he was in the laboratory practicing the tracking task, and he did not realize he was only dreaming that he was doing the task until after he woke up. Analysis of this record indicated that it had 0 saccades during the tracking motion, and that both metrics of smooth pursuit during non-lucid circle tracking were within the 90% prediction interval of lucid REM sleep dreaming trials and were outside the 90% prediction interval for imagination trials. These data suggest that one need not be aware of the fact that one is dreaming (lucid) in order for the imagery of REM sleep dreaming to guide smooth pursuit behavior. This is consistent with other research, which has observed that repeated voluntary saccadic left–right eye movement signals show no observable differences in shape or amplitude in lucid and non-lucid REM sleep dreams^11^. Together, these data suggest that the explicit consciousness of the fact that one is dreaming is not likely to be the determining factor for the oculomotor behavior of either saccadic or pursuit eye movements during REM sleep dreaming and support the interpretation that lucid and non-lucid dreaming are largely continuous with respect to these processes.

Altogether, the present study illustrates the potential of lucid dreaming as a paradigm for the study of consciousness in general and REM sleep dreaming in particular. Experienced lucid dreamers are capable of exercising volitional control over their actions while dreaming and are therefore able to conduct experiments from within EEG-verified REM sleep dreams. This methodology helps to overcome the shot-in-the-dark approach of traditional psychophysiological studies of REM sleep dreams^29^, which relies on collecting large numbers of recordings and extracting small subsets of data in which the content of interest appears by chance. In conclusion, lucid dreaming presents a methodological paradigm that has the potential to open new ways of studying the relationship between consciousness and neurophysiological processes using the dreaming brain as a model system.

## Methods

### Participants

Seven participants (4 men, 3 women) participated in the study (age range = 20**–**48 years). Participants were selected for excellent dream recall (recalling at least one dream a minimum of 5–6 nights per week) and high reported frequency of lucid dreams (3–4 lucid dreams per week minimum, or approximately one lucid dream every other night), with a demonstrated ability to successfully have lucid dreams in a sleep laboratory setting. As there was no pre-specified effect size for these data, we obtained as many trials from as many participants as possible given experimental constraints (see below). All participants had no history of neurological disorder and had normal or corrected-to-normal vision. The study complied with all relevant ethical regulations for research with human subjects and the study protocol was conducted in accordance with the Declaration of Helsinki. Signed informed consent was obtained from all participants before the experiment, and ethical approval for the study was obtained from the Stanford University Institutional Review Board.

### Procedures

Participants spent between 1 and 8 nights in the laboratory during the course of the study. We scheduled multiple nights (depending on participant availability and availability of sleep laboratory facilities) in order to allow for more chances to successfully complete the tracking task during lucid dreams recorded in the sleep laboratory and in order to obtain as many tracking trials from each participant as possible. On each night, EEG (29 channels), sub-mental EMG and vertical and horizontal EOG were continuously recorded. EEG and EOG recordings were made with a Neuroscan 32-channel SynAMP system with a DC amplifier and sampled at 250–1000 Hz. Eye movement analysis and quantification was performed on DC recordings of the EOG. Sleep staging was performed offline using standard criteria of the AASM (American Academy of Sleep Medicine).

### Lucid dream eye signaling and detection

Participants were instructed to mark the moment of initiation of lucidity as well as the initiation and completion of the tracking tasks with volitional LRLR eye movement signals^7^ (referred to as “LRLR”), in which they rapidly moved their eyes all the way to the left then all the way to the right two times consecutively. As demonstrated by LaBerge^12^ these LRLR eye signals are clearly discernable in the electrooculogram (EOG), and can be used to provide objective evidence of lucidity as well as precise time-stamping of tasks and events during lucid REM sleep (for replications and extensions see, e.g., refs. ^9,13^; for recent implementations see, e.g., refs. ^10,15^). The instructions provided to participants were as follows: “When making an eye movement signal, we would like you to look all the way to the left then all the way to the right two times consecutively, as if you are looking at each of your ears. Specifically, we would like you to look at your left ear, then your right ear, then your left ear, then your right ear, and then finally back to center. Make the eye movements by only moving your eyes (without moving your head), and make the full left-right-left-right motion as one rapid continuous movement without pausing.” The instruction to look at each ear is critical, as it encourages full-scale eye movements without corresponding head movements. Detection of lucid dream eye movement signals was performed with a custom algorithm that used template matching and detection of successive L–R–L–R patterned saccades. 100% of the detected signals were subsequently validated by two expert judges (S.L. and B.B.), with an inter-rater reliability of 100%.

### Tracking tasks

Participants performed either circle tracking or 1D horizontal meridian line tracking in each of three conditions: (i) lucid REM sleep dreaming (“dreaming”), (ii) awake with eyes open (“perception”) and (iii) tracking the imagined movement while awake with eyes closed (“imagination”). Additionally, one participant completed a fourth condition in which she imagined tracking the movement in a lucid dream (“imagination in dreaming”). Participants completed the perception and imagination tracking tasks before the overnight sleep recording during the same study visit. Participants were instructed to track the movement using only their eyes (not to move their head), to refrain from blinking until the completion of the tracking motion, and to make the movement slowly. They were instructed to spend approximately 1 s tracking each quarter of the movement. Participants time-stamped the beginning and end of each tracking trial with LRLR eye movement signals. The instruction for the circle tracking task was as follows: “Signal with LRLR; extend your dominant hand at arm’s length with your thumb up, follow the movement of the thumb nail as it moves clockwise in a circle centered in the visual field; signal with LRLR; track a circle counterclockwise; and signal with LRLR.” The instruction for the line task was identical to the circle tracking, with the exception that the task was to “follow the tip of your thumb as you move your hand to the far left, then to the far right passing center, and then back to center.” The tracking instructions were identical for all three conditions (dreaming, perception and imagination) with the exception that for the imagination condition the instruction was to “imagine extending your dominant hand” and to “follow the movement of your imagined thumb”.

### Dream report methods and analysis

Upon awakening, participants were interviewed by the experimenter as to (i) whether they had a lucid dream (YES/NO) and (ii) whether they completed the tracking task of visually tracking the thumb of their hand in the dream (YES/NO). For dreams in which participants reported that they completed the task, they filled out a dream report form in which they wrote a full report of the dream. Participants were instructed to describe in detail the narrative of the dream, including the sequence of events and detailing any thoughts, feelings or sensations that they experienced, as well as how they knew they were dreaming (e.g., if it was triggered by a particular event in the dream). Following standard procedures (i.e., ref. ^7^), lucid dreams were validated through the convergence between phenomenological reports of lucidity and objective eye movement signals in the EOG with concurrent EEG/polysomnography evidence of REM sleep (see “Procedures and Lucid dream eye signaling and detection” above for details on the eye signaling methodology). Reports were also judged for evidence that participants performed the smooth tracking task in the instructed manner (by tracking visual imagery in their dream) by two independent judges. In order to meet this criterion, reports had to include a description of a subjective experience of performing the tracking task during the dream (i.e., “I traced a circle by following my thumb”; “I put out my thumb and followed it down to the right counterclockwise”) (example excerpts of dream reports are provided in Supplementary Note 1).

### Eye movement analysis

Raw EOG signals were converted to angular degrees according to pre- and post-sleep calibrations, baseline corrected and denoised using a 25-pt (100 ms) median filter, which provides optimal noise reduction of the EOG signal^30^. Onsets and offsets of tracking trials were manually marked by a researcher blinded to condition by plotting the HEOG and HEOG vs. VEOG signals between the offset of the initiation LRLR signal and the onset of the completion LRLR signal and removing any data points preceding or following the tracking movement tasks. Automated classification of the EOG during tracking tasks was performed using Velocity and Movement Pattern Identification (I-VMP) implemented in Eye Movement Classification for Matlab, a validated algorithm that classifies saccades, fixations and smooth pursuit eye movements^31–33^. The classifier first identifies saccades using a velocity threshold; signals exceeded this threshold (i.e., 70°/s) that also meet minimum amplitude and duration criteria are classified as saccades. Subsequently, movement patterns in the remainder of the EOG signal are analyzed to classify smooth pursuit movements and fixations. Movement patterns are analyzed in a temporal window (*T*_w_) by computing a set of angles of the eye positional samples in the window. Specifically, lines of best fit to the data points in the window are computed for each set of eye position points in the window in increasing order (*p*_1_ + *p*_2_; *p*_1_ + *p*_2_ + *p*_3_; *p*_1_ + *p*_2_ + *p*_3_ + … *p*_*i*_) and the angles of dispersion between each fitted line are calculated. During pursuit eye movements the dispersion angles tend to follow a linear trend^33^. The magnitude of the movement is then calculated by the sum of angles pointing in a specific direction. In the implementation used here^32^, the magnitude of movement is normalized between 0 and 1 and values are separated with a range threshold *T*_m_. Values exceeding *T*_m_ are classified as pursuit and values below *T*_m_ are classified as fixations. From the segmented EOG, we then computed two scale-free metrics of smooth tracking behavior: (i) number of saccades per second (NSPS), and (ii) the pursuit ratio (SP%), which quantified the amount of time in smooth pursuit divided by the total time spent tracking the movement (SP% = (*T*_pursuit_/*T*_total_) × 100). Parameter values for the classification algorithm were selected based on recommended thresholds^32^. Saccades met three criteria: amplitude ≥4°, velocity ≥70°/s and duration ≥20 ms. I-VMP classification used a range threshold value of 0.2 and temporal window size of *T*_w_ = 0.12 s. Additionally, total circle tracking time had to exceed a threshold of *T*_t_ = 2.0 s. Trials with a total duration less than 2 s (waking: *N* = 0, dreaming: *N* = 1, imagination: *N* = 3) were discarded prior to analysis based on pre-established criteria to exclude the possibility that subjects engaged in eye movements other than slow tracking, as tracking time less than 2 s exceeds the 30°/s velocity threshold for smooth pursuit eye movements with a circle diameter of approximately 30° used here^2^.

### Statistical analysis

Linear mixed models were used in analysis to account for repeated measures with varied numbers of repeated observations within subjects. The model used restricted maximum likelihood estimation, and included participant (SUB) as a random effect, and SESSION, STATE (perception, dreaming, imagination), and (for the circle tracking task) tracking direction (DIR) (clockwise, counterclockwise) as fixed effects. Shapiro–Wilk tests indicated that both NSPS and SP% significantly deviated from a normal distribution (NSPS: Shapiro–Wilk’s *W* = 0.77, *P* *<* 10^−10^; SP%: Shapiro–Wilk’s *W* = 0.85, *P* *<* 10^−7^). We therefore used nonparametric bootstrapping for significance tests. Hypothesis testing of regression coefficients (pairwise tests) from the mixed models was obtained by the following steps: (i) constructing a model based on the null hypothesis of no differences between STATE (*H*_0_); (ii) resampling with replacement the distribution of the response residuals, reconstructing a bootstrap *y* response vector by adding the resampled residuals to *y*, and refitting the *H*_1_ model to the bootstrap response vectors to generate 100,000 bootstrap estimates of the regression coefficients (*β*) under *H*_0_; and (iii) comparing the observed value of the test statistic against the bootstrap distribution under *H*_0_ (two-tailed frequentist *P* value). The 95% CIs on regression coefficients were obtained by computing 1000 bootstrap estimates of the parameter through resampling the residuals under the *H*_1_ model and computing the 2.5% and 97.5% values (frequestist CI). Mixed model construction and mixed model bootstrapping were performed with the lme4 package^34^ in the R environment (R Development Core Team, 2006). Mixed model fixed effects were assessed by means of a bootstrap likelihood ratio test on mixed effects models (PBmodcomp in R) specified with maximum likelihood estimation. ROC analysis was performed using the pROC package in R^35^. Differences in AUC were assessed using a two-tailed bootstrap significance test with 100,000 bootstrap replicates.

### Bayesian classification

Classification of STATE (perception, dreaming, imagination) used a Bayesian classification procedure developed and validated by Allen et al.^36^. The primary advantages of this classification procedure are that it captures within-participant variation and that it allows combination of multiple response indicators to improve classification accuracy. We performed a two-step split-half classification. We first randomly partitioned the dataset into two halves. In the first half, we computed the within-participant *z*-scores for each indicator (predictor variable), and determined the *z*-score cut-point across the dataset that maximized sensitivity and specificity for each indicator. In the second half of the dataset, we then combined the multiple indicators in a Bayesian fashion to calculate the BPP (i.e., the probability that a tracking trial was either from perception or REM sleep dreaming (considered separately) as opposed to imagination given an observed combination of indicators (i.e., high SP% and low NSPS)). Conceptually, the probability ratio is equal to the proportion of tracking trials elicited by perception or dreaming, respectively, that show the combination of indicators divided by the proportion of all trials showing the combination of indicators (1.000 = perfect classification).

### Data availability

The data that support the findings of this study are available from the corresponding author on reasonable request.

## Electronic supplementary material

Supplementary Information
