# Supplementary material for: Smooth tracking of visual targets distinguishes lucid REM sleep dreaming and waking perception from imagination
Source: Nat Commun. 2018 Aug 17;9:3298. doi: 10.1038/s41467-018-05547-0 (PMC6098118; doi:10.1038/s41467-018-05547-0)
Supplement: Supplementary file 1 — Supplementary Information [file 41467_2018_5547_MOESM1_ESM.pdf]

Supplementary Information

Smooth tracking of visual targets distinguishes lucid REM sleep dreaming and waking perception from imagination

LaBerge et al.

Supplementary Figure 1

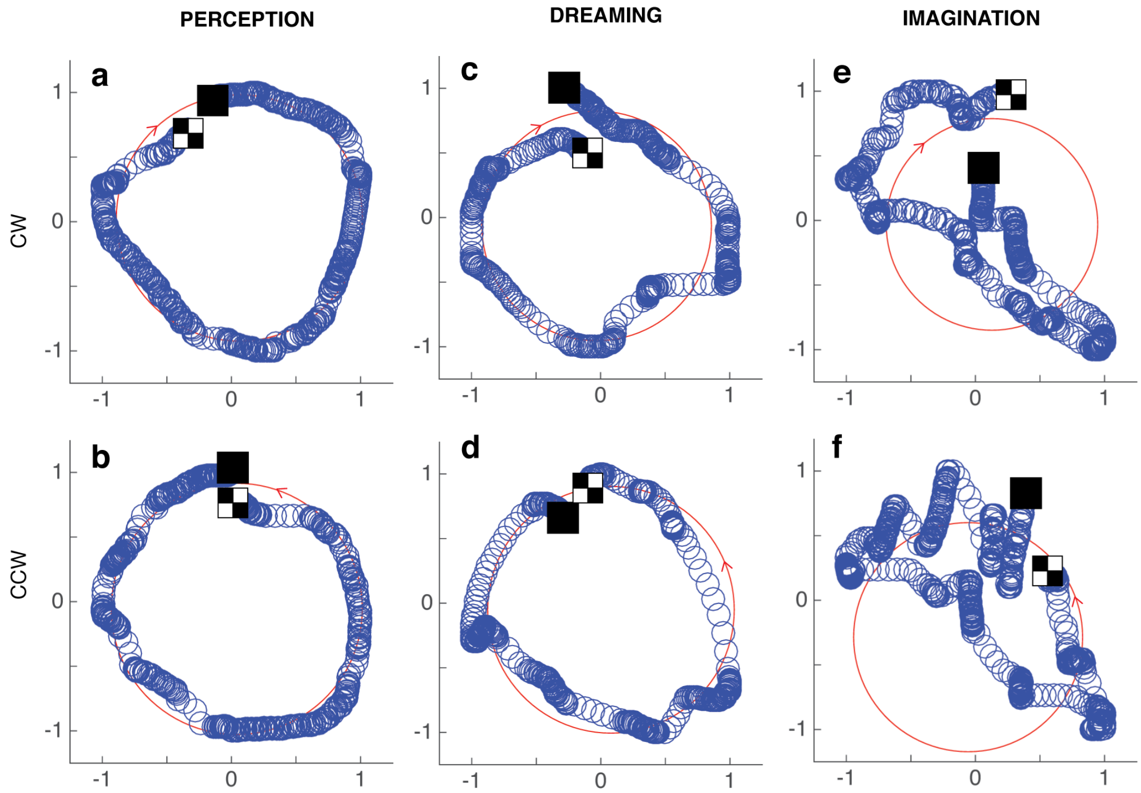

Supplementary Figure 1. Circle tracking in a representative subject. Electrooculogram tracking of slow circular movement during perception (eyes-open, waking state) (**a,b**), dreaming (eyes-closed, REM sleep) (**c,d**), and imagination (eyes-closed, waking state) (**e,f**) in a representative subject. Black squares designate start of tracking, checkered squares designate end of tracking. Red circles show the best fitting circle minimizing least squares error to the normalized tracking motion. Red arrows indicate direction of tracking. CW = clockwise tracking; CCW = counterclockwise tracking.

Supplementary Figure 2

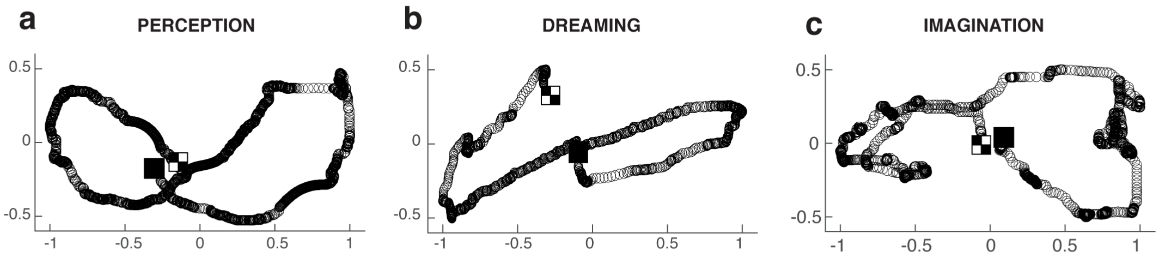

Supplementary Figure 2. Lemniscate tracking. Eye tracking of lemniscate (infinity sign) during perception (**a**), REM sleep dreaming (**b**), and imagination (**c**). Black squares designate start of tracking, checkered squares designate end of tracking.

Supplementary Note 1: Examples of participant reports of completing the smooth tracking task during lucid REM sleep dreams

Report of line tracking task (Participant 3; Figure 1)

“I was lying in bed feeling relaxed when I decided to try and dream. I knew I was dreaming as soon as the dream started. I found myself standing in a booth in the dream. I paused, standing still, thinking that I should do the experiment. At first I decided not to do the experiment because it was too dark in the dream booth. I went to the booth door and noticed some lights shining through the cracks. I opened the door. My uncle was sitting in a chair to my left, although I could only see the back of his head. I said, “Hi, James.” I think he answered, “Hi,” but I am not sure. I stood beside him hoping that he wouldn't interfere. ***I recalled to do the line task. I gave a beginning left-right-left-right (LRLR) signal, then I stretched my arm out and stuck my thumb up so that I could see it. Tracking my thumb with my eyes, I moved my hand to the far left, then to the far right passing center, and then back to center. When I was tracking to the right, I noticed I turned my head a little to the right and back to the center when I began tracking back to the left. The whole tracking took approximately 10s. During the tracking, I worried that I might wake up before I could finish the experiment. I gave an ending LRLR signal.*** Next, I walked out into the hallway and looked to my left down the hallway wondering what else I should do. I saw some boxes or something about 20 feet away. I decided to fly into the air and down the hallway. As I flew past the boxes, I decided I would yell out to all my dream characters to stop me if they wanted to talk with me. I came upon two dream figures who were walking down the hallway as I was yelling out my message repeatedly. These two ignored me. Then I flew into a cafeteria area where a woman to my right said she wanted to talk with me and for me to come with her…”

Report of circle tracking task (Participant 1; Supplementary Figure 1)

“I’m coming home to my mother’s place. The door to the garden and the mailbox are strange; the mechanisms to close them are too complicated. I think: ”It’s too complicated, that’s strange… hey, this could be a dream!” To confirm, I jump into the air and keep levitating, although I was already pretty sure about the fact that I was dreaming before that dream test. I’m pretty excited, say aloud ”This is a dream!” and very soon thereafter ***eye signal left-right-left-right (LRLR). I leave the garden, go into the street, and I’m thinking: “Hopefully I don’t wake up when I perform the experiment!” My vision is not perfect, but I start anyway: I signal left-right-left-right, then trace a circle by following my thumb counterclockwise, signal again left-right-left-right, then trace the clockwise circle.*** After I finished the circles, I think: “Great, now I can do whatever I want, the experiment is done.” … I walk down the street, and over there is the beach. It’s pretty dark now. I throw away my glasses and some clothes. A short resistance (I won’t find all that again), but, hey, it’s a dream! I think: ”Swim with the dolphins!” What’s in the water, though, turn out to be lots of monsters. Hippopotamus-like ones, and others

that are more fantasy-like. I think: ”I have to face them, hopefully figure out what they represent and befriend them!...”

Report of lemniscate (infinity sign) tracking task (Participant 4; Supplementary Figure 2)

“In my dream I was looking down a room to where a dark-haired lady was walking away from me to or through a hall. I realized I was dreaming and got very excited (being careful not to wake myself up), because I knew it was *the* dream in which I could do the experiment. ***I stopped, stood still and moved my eyes left-right-left-right very distinctly. I slowly raised my right hand, put out my thumb and followed it down to the right counter clockwise up and around as I had done before sleeping two times. Then as I did the LRLR eye signals again I was so happy that I did it all correctly and also stayed in the scene.*** I seemed to be on a hospital floor with woman workers or nurses mostly sitting at tables or stations and doing some kind of work. I decided to explore and walked to where more people were. I passed a large mirror wall and saw my reflection. This was fun and funny. I didn’t look as bad as I imagined and said so to a light-haired woman sitting to my right. Looking at my reflection, I had less electrodes on than when I looked in the Stanford bathroom before going to sleep. I also looked thinner and was wearing a gauzy night gown even though I slept in black pants and a tee shirt…”

Supplementary Table 1. SPEM X STATE for circle tracking and 1D horizontal meridian tracking

	State	*Circle*		*1D meridian*	
		Mean	*SD*	*Mean*	*SD*
SP%	P	90.03	5.25	95.18	2.62
	D	87.02	11.47	93.66	5.46
	I	64.76	15.10	82.55	1.67
	IinD	-	-	65.95	16.18
NSPS	P	0.15	0.23	0.08	0.14
	D	0.62	0.85	0.09	0.11
	I	1.98	1.14	1.01	0.47
	IinD	-	-	1.34	0.70

* *SPEM* = smooth pursuit eye-movements. *NSPS* = number of saccades per second. SP% = pursuit ratio.

Supplementary Table 2. Single subject SPEM X STATE for circle tracking

		Dreaming			Perception		
	Participant	*beta*	*z*-diff	*n*	*beta*	*z*-diff	*n*
SP%	1	35.5	2.09	5	30.0	1.62	4
	2	6.58	0.80	7	11.6	1.34	8
	3	34.9	1.42	6	35.6	1.84	12
	4	18.04	2.20	1	13.09	1.51	2
	5	1.66	0.13	2	20.31	1.65	2
		∑(z-diff):	6.51		∑(z-diff):	7.95	
		∑(z-diff)/√N:	2.91		∑(z-diff)/√N:	3.56	
NSPS	1	-2.53	-1.75	5	-2.67	-1.76	4
	2	0.15	0.14	7	-0.83	-0.95	8
	3	-2.38	-1.22	6	-2.34	-1.83	12
	4	-1.13	-1.63	1	-1.42	-1.77	2
	5	-0.77	-0.74	2	-1.59	-1.53	2
		∑(z-diff):	-5.83		∑(z-diff):	-7.65	
		∑(z-diff)/√N:	-2.61		∑(z-diff)/√N:	-3.42	

**SPEM* = smooth pursuit eye-movements. *NSPS* = number of saccades per second. *SP%* = pursuit ratio. *beta* = regression coefficients. *n* = number of smooth tracking task epochs. *N* = sample size. *p*-values and *z*-diff show pairwise comparisons to imagination condition (2000 bootstrap estimates).

Supplementary Table 3. Single subject SPEM X STATE for 1D horizontal meridian tracking

		Dreaming			Perception		
	Participant	*beta*	*z*-diff	*n*	*beta*	*z*-diff	*n*
SP%	3	7.99	1.28	3	14.28	2.21	2
	4	14.7	1.98	1	9.38	1.26	1
		∑(z-diff):	3.26		∑(z-diff):	3.47	
		∑(z-diff)/√N:	2.31		∑(z-diff)/√N:	2.45	
NSPS	3	-1.25	-1.91	3	-1.27	-2.18	2
	4	-0.47	-1.99	1	-0.23	-0.97	1
		∑(z-diff):	-3.90		∑(z-diff):	-3.15	
		∑(z-diff)/√N:	-2.75		∑(z-diff)/√N:	-2.23	

* *SPEM* = smooth pursuit eye-movements. *NSPS* = number of saccades per second. SP% = pursuit ratio. *beta* = regression coefficients. *n* = number of smooth tracking task epochs. *N* = sample size. *p*-values and *z*-diff show pairwise comparisons to imagination condition (2000 bootstrap estimates). One additional trial (n=1) was obtained from another participant (SW) only in the REM sleep dreaming condition. Therefore, single-subject statistics between states (beta and z-diff), could not be calculated for this participant.

Supplementary Table 4. Bayesian classification during circle tracking

Indicators	Perception		Dreaming		Z-Score Cutpoint
	Sensitivity	Specificity	Sensitivity	Specificity	
Z SP%	1.00	0.93	0.75	0.93	-0.29
Z NSPS	0.94	0.93	0.75	0.93	-0.11

Participant	Bayesian Posterior Probabilities	
	Perception	Dreaming
Total	0.996	0.989
1	1.000	1.000
2	0.960	0.800
3	1.000	1.000
4	1.000	1.000
5	1.000	0.000[†]

* *Z NSPS* = within-participant z-transform of number of saccades per second. Z SP% = within-participant z-transform of pursuit ratio. Bayesian posterior probabilities show the probability that a tracking trial is classified as from perception or dreaming compared to imagination (1.000 = perfect classification) combining the multiple indicators of Z SP% and Z NSPS. [†] This estimate is based on only one trial in the split-half analysis.

Supplementary Table 5. Bayesian classification during 1D horizontal meridian tracking

Indicators	Perception		Dreaming		Z-Score Cutpoint
	Sensitivity	Specificity	Sensitivity	Specificity	
Z SP%	1.00	1.00	0.75	1.00	-0.29
Z NSPS	0.67	1.00	1.00	1.00	-0.11

Participant	Bayesian Posterior Probabilities	
	Perception	Dreaming
Total	1.000	1.000
3	1.000	1.000
4	1.000	1.000

* *Z NSPS* = within-participant z-transform of number of saccades per second. Z SP% = within-participant z-transform of pursuit ratio. Bayesian posterior probabilities show the probability that a tracking trial is classified as from perception or dreaming compared to imagination (1.000 = perfect classification) combining the multiple indicators of Z SP% and Z NSPS. One additional trial (n=1) was obtained from another participant (SW) only in the REM sleep dreaming condition. Therefore, single-subject classification between states was unable to be calculated for this participant.
